# The e-RFIDuino: An Arduino-based RFID environmental station to monitor mobile tags

**DOI:** 10.1016/j.ohx.2021.e00210

**Published:** 2021-06-12

**Authors:** Mathieu Cassel, Oldrich Navratil, Franck Perret, Hervé Piégay

**Affiliations:** a University of Lyon, UMR 5600 CNRS-Environnement Ville Société, ENS de Lyon, 15 Parvis René Descartes, Lyon Cedex, France; b University of Lyon, UMR 5600 CNRS-Environnement Ville Société, University Lumière Lyon 2, 5 avenue Pierre Mendès-France, Bron Cedex F-69635, France

**Keywords:** Power environmental station, Active UHF transponders, Object monitoring, Bedload, Floating wood, Environmental RFID monitoring station

## Abstract

We present a datalogger based on Arduino cards and commercially available tools for radio frequency identification, which we term the e-RFIDuino. Designed to be robut, easy to build and install, it detects and records the mobility of objects tagged with active transponders emitting in the ultra-high frequency domain (433.5 MHz). It functions without connection to the power supply network and is adapted to harsh outdoor environments. Once installed in the field and its on-site sensing field is determined, the data collected (timestamp of detection, transponder identification number, and received signal strength indication) allow estimation of the virtual velocity of tracer passage and investigation of displacement patterns at the scale of the area of detection. Experimental tests showed the device to have very high effectiveness when used to monitor the passage of sediment tracers in a torrential river system during various flood events over several months. The total cost to construct this open source device is below 850 Euros, and it is easily customizable. In the future, it could be equipped with a system for data transmission over the mobile telephone network to reduce the field effort and time required to obtain data, and to provide real-time triggering of field acquisitions at the most appropriate times.

## Graphical abstract

**Figure:**
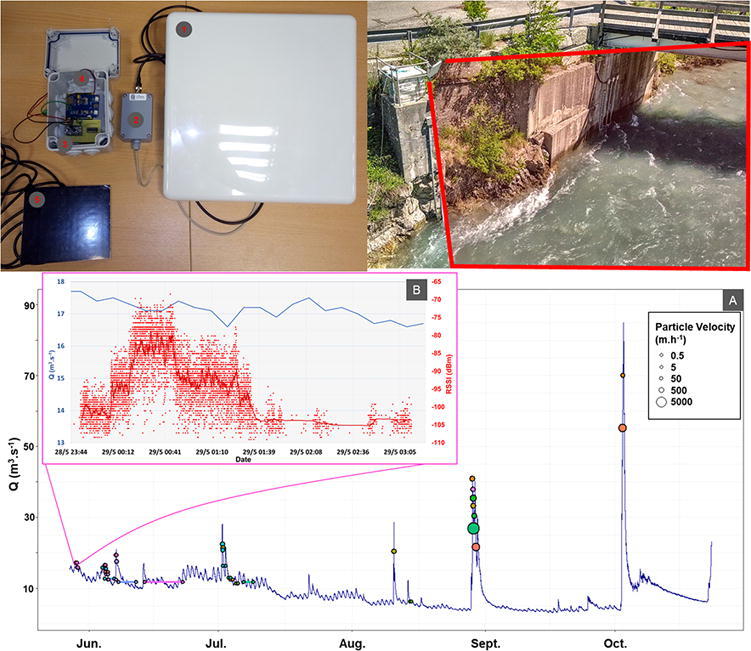

## Specifications table

**Table:** 

Hardware name	*e-RFIDuino: an autonomous Transponder Datalogger*
Subject area	• Engineering and Material Science • Environmental, Planetary, and Agricultural Sciences • Educational Tools and Open Source Alternatives to Existing Infrastructure • General
Hardware type	• Field measurements and sensors • Mechanical engineering and materials science
Open Source License	Creative Commons Attribution 4.0 International license
Cost of Hardware	*< 850 Euros*
Source File Repository	Reserved DOI: https://doi.org/10.17632/tkfj9j7z2m.1

## Hardware in context

Transponders for radio frequency identification (hereinafter referred to as RFID tags) have been widely used to monitor the displacement of mobile objects (such as coarse sediment particles [1], [2] and wood pieces [3], [4]) and wildlife species (such as fish [5], [6], [7]) in riverine or coastal environments. The choice of one tracking technique depends on the users’ study objectives, and is determined by a set of factors including data requirements, costs (equipment and follow-up), experiment duration, and the characteristics (e.g. water depth, velocity, total area concerned) of the studied site [8]. Recent investigations in river bedload transport and coarse sediment tracking using active ultra-high frequency transponders (hereinafter referred to as a-UHF tags) have led to substantial technical and methodological developments, including the surveying of tracked objects by unmanned aerial vehicles (UAVs) [8].These developments have profited from three properties of the a-UHF RFID system [9]:

- a signal anti-collision algorithm that allows the individual detection of several tracers when they are simultaneously present within the antenna sensing field;
- a received signal strength indication (RSSI) that allows the accurate geolocation of these objects according to 2D spatial interpolations;
- a semi-directive sensing field antenna that allows the detection of buried or immersed tagged objects over depths of several meters and the establishment of intelligent survey protocols based on interpretations of variations in RSSI.

Unlike the passive integrated transponders (called PIT tags) widely used in sediment tracking studies and functioning without electric battery, the a-UHF tags are battery powered. The COIN ID tag model we used, developed by the ELA Innovation Company, is distributed by the CIPAM Company. It is emitting a beacon signal in ultra-high frequency domain (433.9 MHz) at a maximal power emission of 22 µW with a vertical linear polarization whereas PIT tag antenna emit in low frequency (134.2 kHz). It consists of an electronic chip of cylindrical shape with a thickness and diameter of 7 mm and 24 mm respectively, with an −16.56 Db gain antenna at its base. It is packed in a waterproof housing (IP 68) that has a thickness and diameter of 11.5 mm and 36 mm, respectively. Its operating life depends on the signal impulse interval selected by the user (about 36 months when emitting every 2.2 s). The a-UHF tags signal is demodulated by a reader communicate via a proprietary protocol called SCIEL®. Also, unlike the PIT tag readers classically used, operating in cycles of approximately 0.09 s divided into charging, listening/reading and synchronization phases, the SCIEL reader is only reading. It has a receiver sensitivity of – 107dBm.

Using this a-UHF RFID technology, we developed an environmental RFID station (hereinafter referred to as the “e-RFIDuino”) to detect and timestamp tracked objects, which in this example are artificial pebbles[10], [11]. Although previous investigations [3], [5], [6], [7], [12], [13], [14] have reported the use of stationary antenna, our system has several significant advantages: it is the first light and complete implementation with a datalogger that is fully automatic (no operator needed after installation), autonomous in power requirements, low-cost, adapted to harsh outdoor environments and little-, if not non-intrusive to the channel stream.

The device was designed to meet the following requirements:

1. Low cost per unit (< €850)
2. Small size
3. Autonomous power supply
4. Weatherproof (IP65)
5. Ease to set up and move
6. Capability to:
  a. detect all the signals emitted by one or several a-UHF transponders present within the antenna sensing field,
  b. timestamp their identification numbers and RSSI
7. Generation of a comma-separated text file with the raw RFID frame, transponder ID, RSSI, and date and time
8. Capability to record files on an easy-to-transfer microSD card
9. Ready availability of components and ease of construction

## Hardware description

**Fig. 1:**
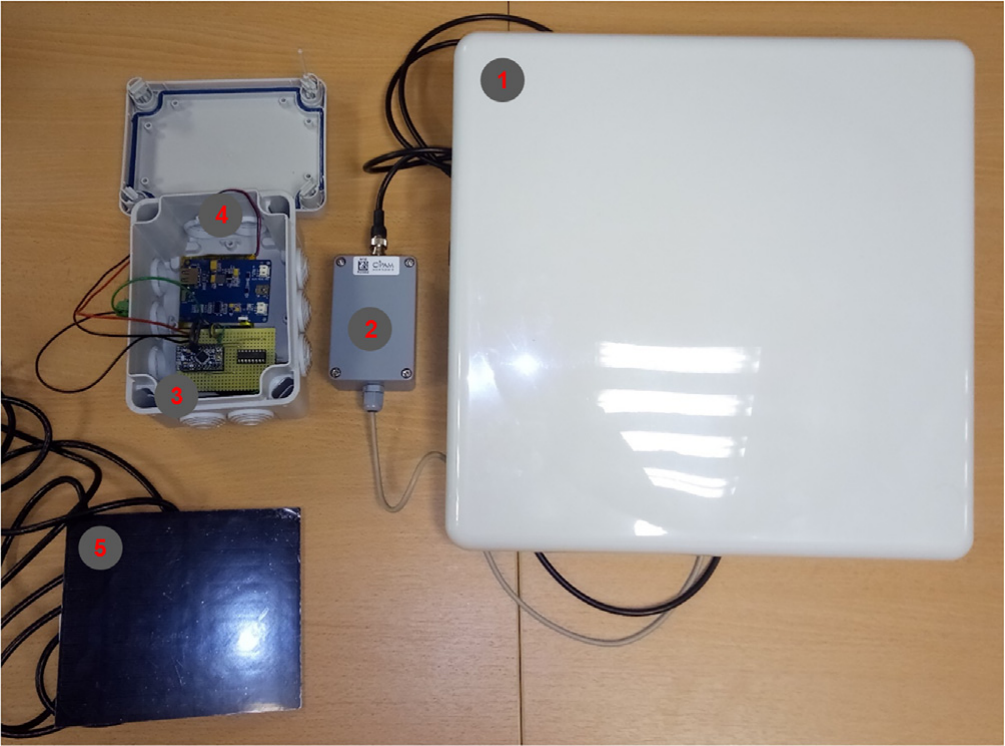
The e-RFIDuino station and its five connected modules: RFID antenna (1), RFID SCIEL reader (2), Arduino datalogger, LiPo Rider, and batteries (3), housing box (4), and small solar panel (5).

**Fig. 2:**
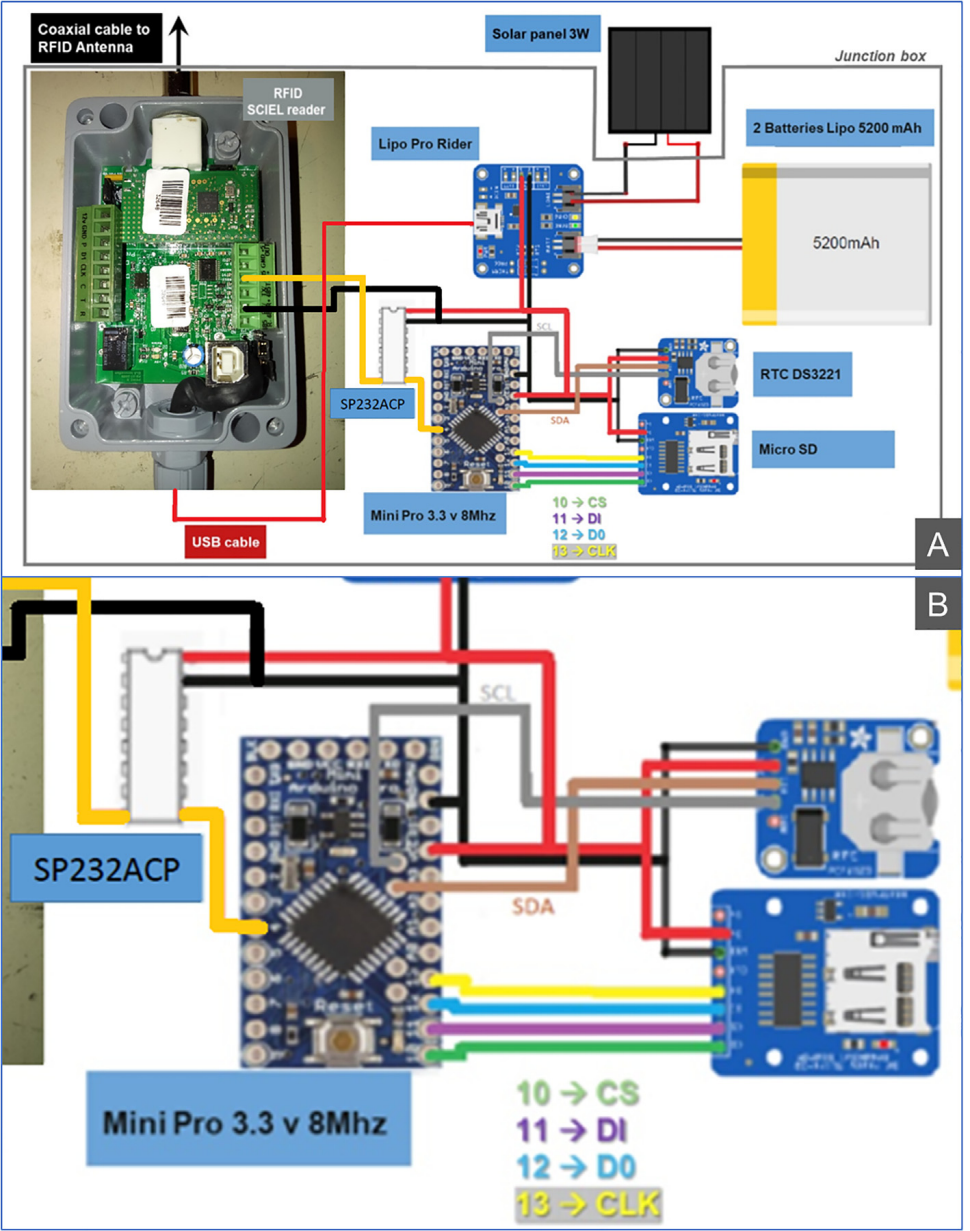
Wiring diagram. (A) Global hardware architecture of the e-RFIDuino station. (B) Focus on the wiring diagram for the Mini Pro, RTC, and MicroSD reader (the wire colors do not correspond to Fig. 5).

The e-RFIDuino station is composed of four connected modules (Fig. 1, Fig. 2):

1. an RFID antenna: Slender III model from the Ela Innovation Company,
2. an RFID SCIEL reader: SCIEL Reader model from the Ela Innovation Company,
3. an Arduino datalogger made up of a Mini Pro card (3.3 V, 8 MHz) with an RTC DS3221 circuit (real-time clock, including a backup battery [CR1220]) and a Micro SD card reader (DEV13743, Sparkfun electronic),
4. a small solar panel (SOL3W, 3Wc, 5.5 V/540 mA, size: 160 × 138 mm) with a LiPo Rider Pro solar panel regulator (SEEED Studio) and two LiPo batteries (3.8 V, 5100 mAh/unit, SEEED Technology Corp.).

The Slender III RFID antenna model was chosen for two reasons. First, it has waterproof (IP 67) a housing and connector designed for harsh outdoor environments with a weight of 2 kg. Second, it is semi-directive which means that it has a cone-shaped sensing field with an aperture angle of 80° and isotropic gain of + 8 dBi. The RFID SCIEL® reader model was chosen because it has a small compact housing adapted to harsh environments (IP65) and is low power consuming (300mWh), which is also adapted for operating outside of power supply network environments. It is ‘field-ready” and does not need specific configuration. It was previously successfully used on different applications including a monitoring trolley [9] and a UAV [8]. The Arduino Minipro is a low-power microcontroller that allows the signal from the RFID reader to be recorded correctly. The RTC is very accurate because it is temperature-compensated, which limits time drifts. The RTC module is powered by a backup battery (CR1220) so that it is not necessary to recompute it if the LiPo batteries are disconnected during maintenance operation or data download. Otherwise, all the other components are standard and easily available.

The connections between the different components are as follows (Fig. 2):

1. The RFID antenna is connected to the RFID reader by a coaxial cable with two BNC connectors, that of the antenna end being waterproof.
2. The output signal of the RFID reader (RS232 transfer protocol) is converted into a TTL (Transistor–Transistor Logic) protocol with an SP232ACP integrated circuit and is then sent to the Arduino datalogger.
3. The power module supplies all the elements.
4. The uploading of the program file into the Mini Pro card from a computer is made via a USB cable and a USB FTDI GT1125 converter.

Finally, a polypropylene junction box (105 × 80 × 150 mm, IP65) houses the RFID reader, the Arduino module, the regulator, and the batteries.

The e-RFIDuino station was designed to handle a large range of applications requiring the monitoring of individually tagged objects, especially in harsh outdoor environments far from the power supply network. The potential applications include a wide range of animals and natural objects that could by identified or distinguished using RFID tags.

When an object under monitoring moves in a single direction (i.e., from upstream to downstream or uphill to downhill), the e-RFIDuino station can be used to detect and timestamp the object entry and exit of the sensing field. The time period during which the object is present within the on-site sensing field of the antenna can be calculated, as well as its overall speed. The timestamp makes it possible to link these observations with other monitored time series (e.g., water discharge or shear stress).

If the objects or animals being monitored are freely moving, it is also possible to estimate their residency duration, number, frequency, and time of passage, and if the detections of a tagged animal are concomitant with those of other individuals, the method could also provide information on wildlife social dynamics and interactions.

## Software description

**Fig. 3:**
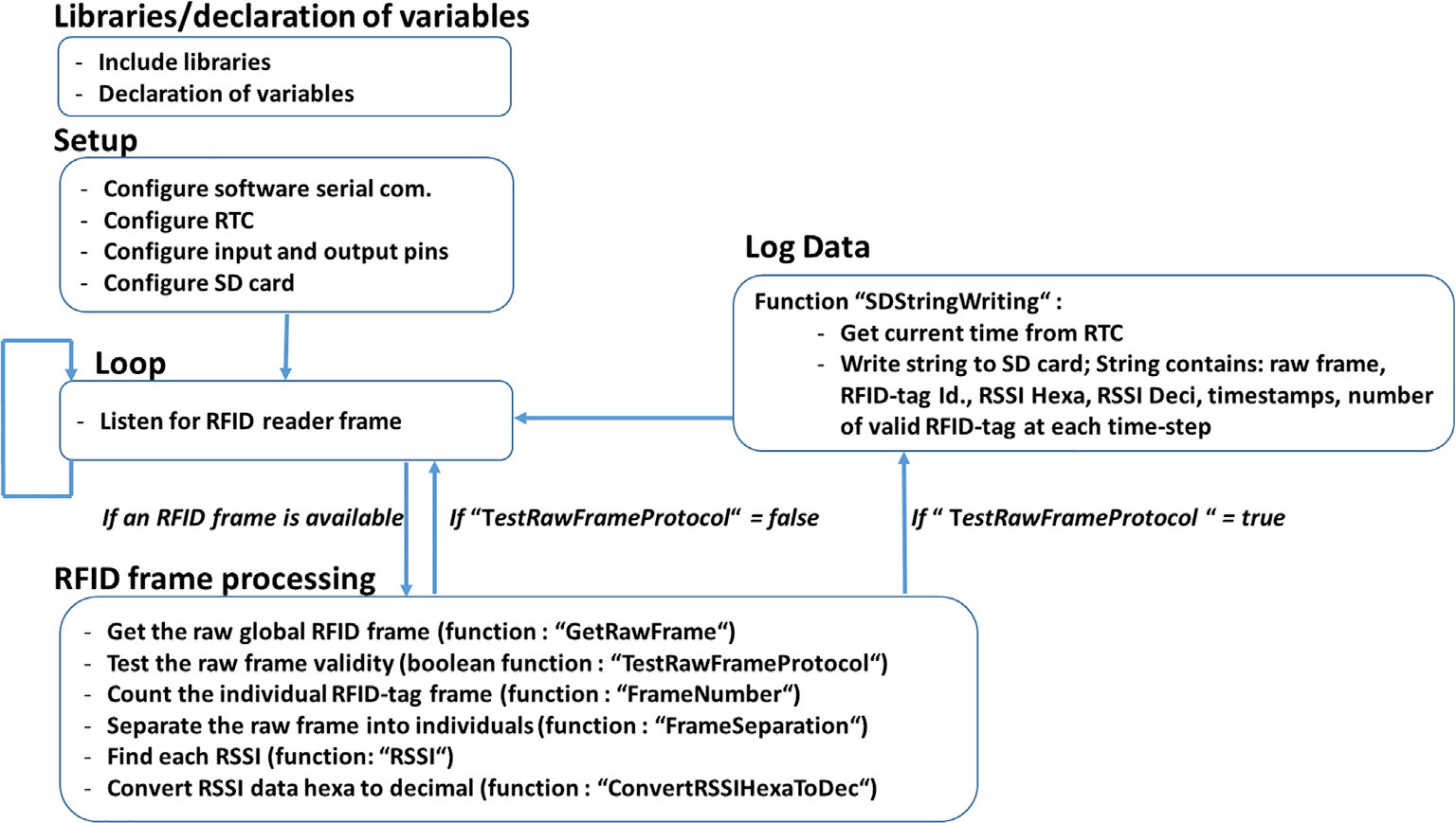
Architecture of the device software with the different functions used.

The software for the e-RFIDuino station was coded on an Arduino IDE platform (downloadable at: https://www.arduino.cc/en/Main/Software). The program consists of a main section and seven functions, and has the following features (summarized in Fig. 3).

**Fig. 4:**
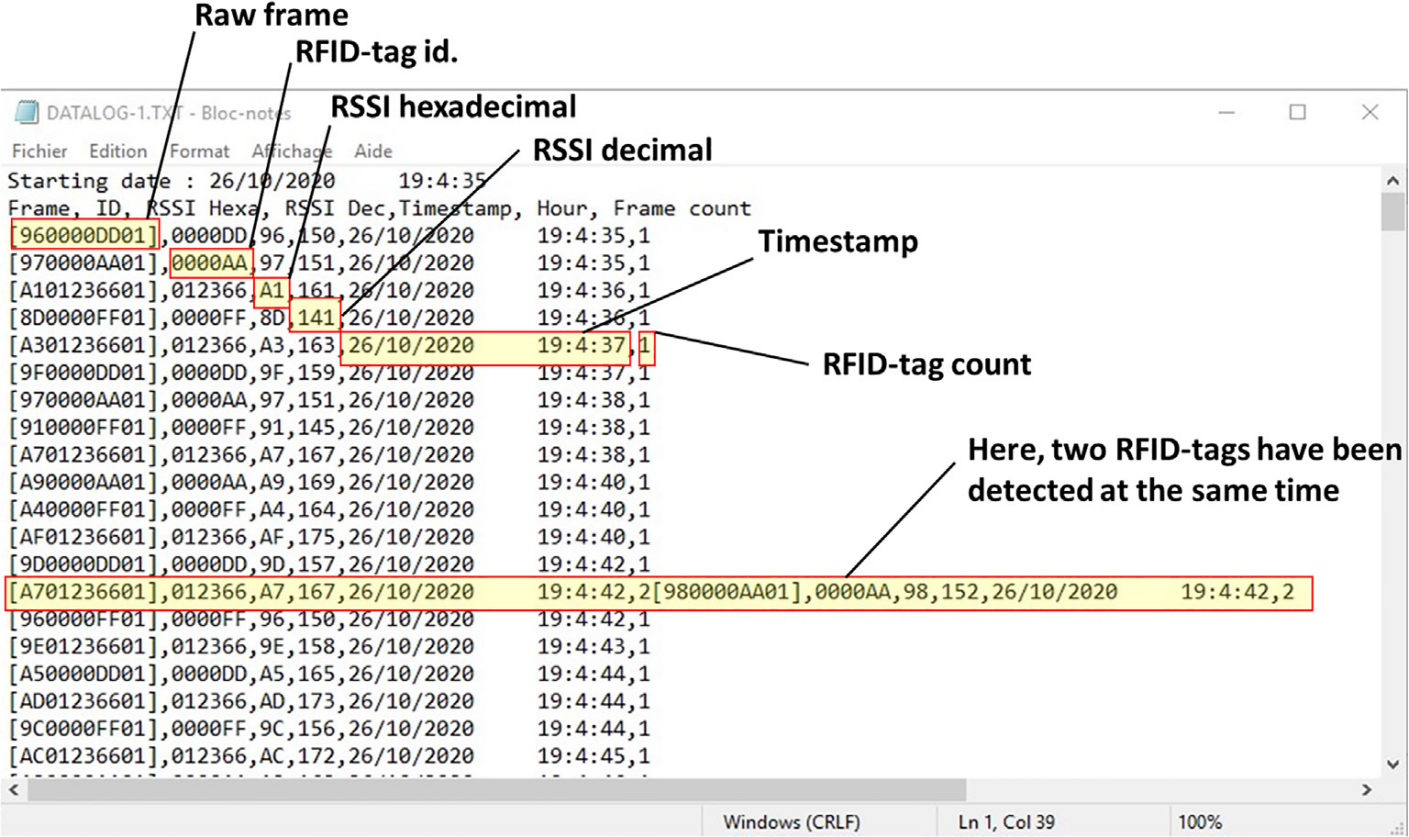
Example output text file.

- The “Libraires and variables declaration”. Different default libraries are used: SPI.h to manage the SPI-bus; SD.h for the SD card; Wire.h to manage the I2C-bus; and SoftwareSerial.h to manage the virtual serial port. Only RTClib.h for the RTC is not available by default for the Arduino IDE platform, and is instead provided by Adafruit Version 1.12.4. Next, all the variables are declared.
- The “Setup” section is used to configure the software serial communication (9600 bauds), RTC, input and output pins, and SD card.
- The “Loop” section listens to the RFID reader. If one or many RFID tags pass into the antenna sensing field simultaneously, a raw RFID frame is created and is then analyzed by the “RFID frame processing” section.
- The “RFID frame processing” section calls six different functions to process the raw RFID frame (GetRawFrame, TestRawFrameProtocol, FrameNumber, FrameSeparation, RSSI, ConvertRSSIHexaToDec).
- If the raw RFID frame is valid (TestRawFrameProtocol function), then at the “Log Data” section, the “SDStringWriting” function obtains the current time from the RTC and writes a string to the SD card. This string contains the raw frame, RFID-tag ID (hexadecimal), hexadecimal RSSI, decimal RSSI, timestamps (day/month/year hour:minute:seconds; dd/mm/yyyy hh:mm:ss) and the number of valid RFID-tags detected at each time-step (or loop in Fig. 3). An example output text file is provided in Fig. 4.

## Design files

Design file 1 corresponds to the Arduino program file, and includes five libraries, a main program, and seven functions (Fig. 3).

### Design file summary

**Table:** 

Design file name	File type	Open source license	Location of the file
Design file 1	INO file	Creative Commons Attribution 4.0 International license	Reserved DOI: https://doi.org/10.17632/tkfj9j7z2m.1

## Bill of materials

Bill of materials

**Table:** 

Designator	Component	Number/quantity	Cost per unit -currency	Total cost -currency	Source of materials	Material type
RFID antenna	RFID Antenna 433 Mhz SLENDER III	1	€198	€198	https://www.cipam.com/produits/ela-rfid-active-101/slender-iii	Other
RFID reader	SCIEL READER RU	1	€399		https://www.cipam.com/produits/ela-rfid-active-101/sciel-reader-r-262	Other
Coaxial cable (Antenna to reader)	soft BNC / N IP67	5 *m*	€58	€58		Other
RS232 Transceiver Ics	SP232ACP; MaxLinear	1	€4.69	€4.69	https://fr.rs-online.com/web/p/transceivers-de-ligne/5403268/	
Battery	Accu Li-Ion 3.8 V 5100 mAh; SEEED Technology Corp.	2	€29.95	€59.90	https://www.gotronic.fr/art-accu-li-ion-3–8-v-5100-mah-5815.htm	
Solar panel	SOL3W; 3Wc; 5.5 V/540 mA − 160 × 138 mm	1	€19.00	€19.00	https://www.gotronic.fr/art-cellule-solaire-sol3w-18996.htm	
Solar panel regulator	LiPo Rider Pro; SEEED Studio; ref: 106,990,008	1	€15.60	€15.60	https://www.gotronic.fr/art-carte-lipo-rider-pro-106990008–19050.htm	
Micro Controller card	Mini Pro Module, 3.3 V / 8 MHz	1	€10.48	€10.48	https://www.robotshop.com/eu/fr/microcontroleur-pro-mini-arduino.html	
Real Time Clock	RTC DS3231 AT24C32 IIC Clock Timer for Arduino	1	€3.99	€3.99	https://www.ebay.fr/itm/RTC-DS3231-AT24C32-IIC-Clock-Timer-Pour-Arduino-Raspberry-Replace-DS1307-Pile/262791426448?hash = item3d2f96f190:g:giEAAOSwIWVY-81Y	
Button cell battery for RTC	Lithium battery CR1220	1	€2.50	€2.50	https://www.gotronic.fr/art-pile-varta-au-lithium-cr1220-19141.htm	
Micro SD Card reader	Sparkfun electronic, DEV13743	1	€7.90	€7.90	https://www.gotronic.fr/art-module-carte-micro-sd-dev-13743–24810.htm	
Micro SD Card	Carte micro SD 8 GB	1	€12.95	€12.95	https://www.gotronic.fr/art-carte-micro-sd-8-gb-21475.htm	
Electronic board	Board Roth Elektronik	1	€8.28	€8.28	https://fr.rs-online.com/web/p/cartes-a-bandes/5185932/	
Computer to Mini Pro card connection (to upload the program)	USB-Series FTDI GT1125 converter and USB cable, Iduino	1	€9.90	€9.90	https://www.gotronic.fr/art-convertisseur-usb-serie-ftdi-gt1125-26140.htm	
Junction box	Polypropylene; 105 × 80 × 150 mm; IP55	1	€21.14	€21.14	https://fr.rs-online.com/web/p/boites-de-derivation/0151741/	

## Build instructions

**Fig. 5:**
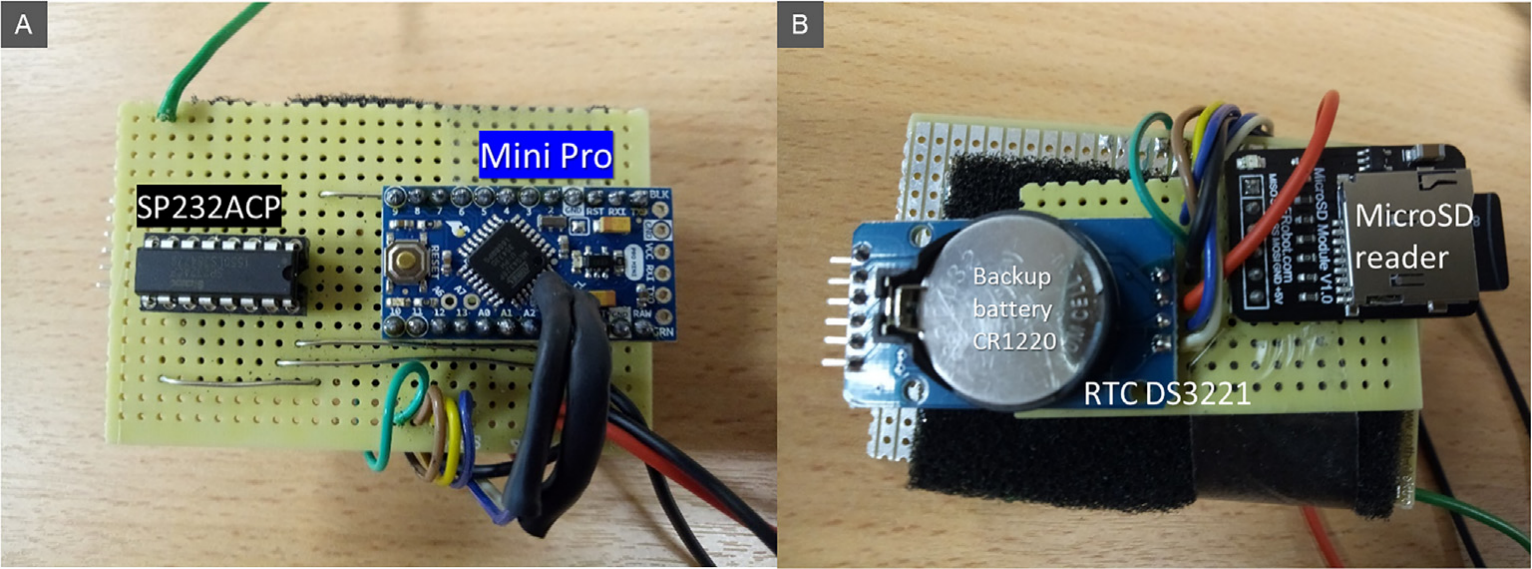
Mini Pro card (A) and MicroSD reader, RTC (B).

Assembly is carried out according to the supplied electronic plan (Fig. 1, Fig. 2, Fig. 5). There is no particular order to respect, nor any particular difficulty.

The only potential safety concern involves the mounting of the two LiPo batteries (5100 mAh; 3.7 V) together in parallel to obtain a single LiPo battery of 10 200 mAh and 3.7 V. Indeed, this step can cause a fire hazard in the event of a short circuit during installation, or different charge levels of the two batteries when connected in parallel. The following procedures must be strictly respected when connecting the two batteries together.

- Before connecting the batteries in parallel for the first time, it is strongly recommended to charge both batteries to their maximum level (approx. 4.2 V). The LEDs must be green when each battery is connected to the LiPo Rider card (switch button on the side of the Lipo card).
- The battery cables must be cut one by one to avoid any short circuit at this stage. Connect the two black wires (GND) together with the black wire of the connector (retrieved from one of the two batteries), solder, and isolate them. Then, strip the red wires and solder them together with the red wire of the connector and insulate the whole. It is important not to create a situation where two stripped wires of opposite polarity can meet.

**Fig. 6:**
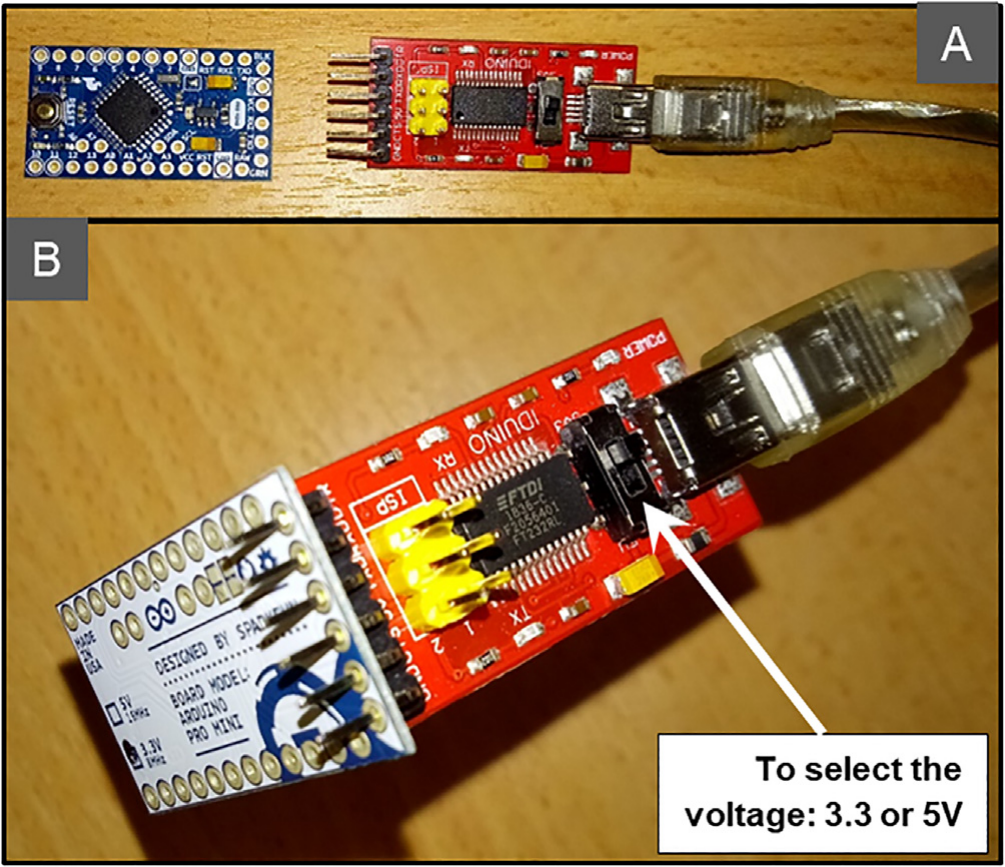
Program upload from PC to Mini Pro using the USB-Series FTDI GT1125 converter. Disconnected (A) and connected (B).

To upload the program to the Arduino board, the host PC needs to be connected to the Mini Pro with a USB-Series FTDI GT1125 converter (Fig. 6). In the Arduino IDE, click on “Tools/Port” and select the desired port. Next, in “Tools/Port” select “Tools/Card type: ‘Arduino Pro or Pro Mini’”. If not available, go into the AVR Arduino boards and Arduino Pro or Pro Mini. In “Tools/Processor”, select “ATmeag328P (3.3 V, 8 MHz)”. The program can then be uploaded. The blue LEDs of the USB-Series FTDI GT1125 converter are blinking.

**Fig. 7:**
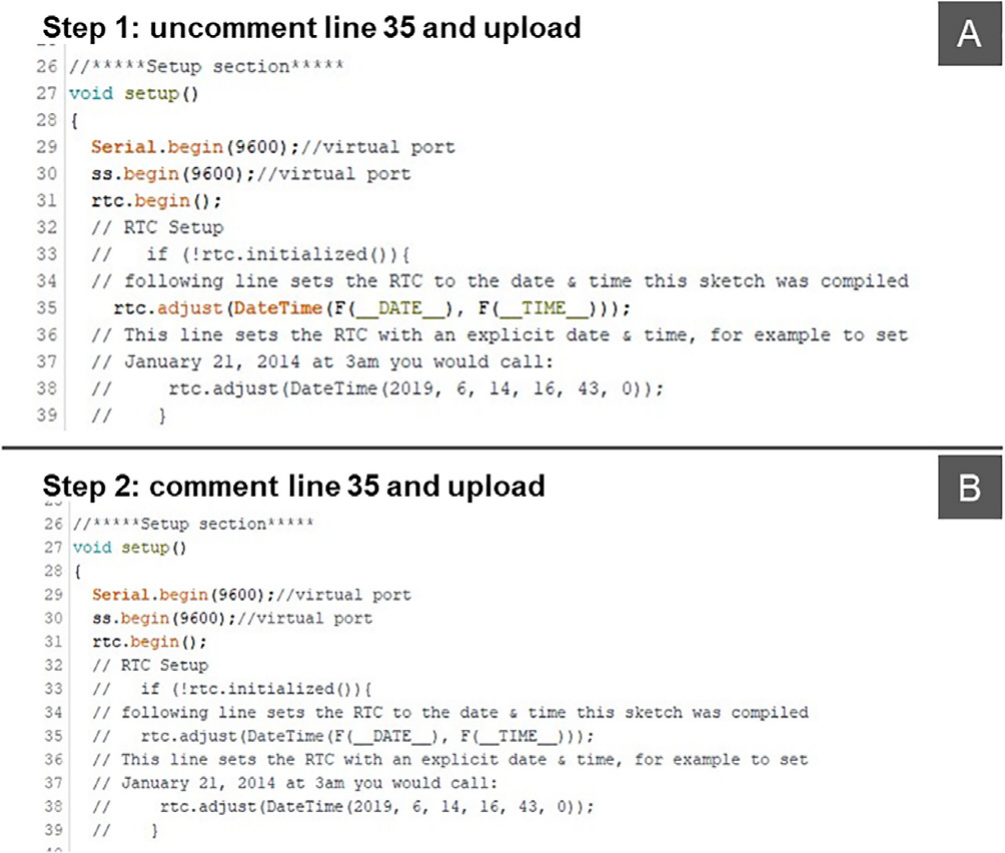
Two-step procedure to set the RTC time.

To set the RTC time automatically when the power is turned on for the first time, or to reset the RTC time if it has drifted, a two-step procedure must be followed (Fig. 7). In step 1, uncomment line 35 to set the RTC clock with the computer time and upload the program file to the Arduino board with the RTC plugged (Fig. 7A). In step 2, comment line 35 and then re-upload the program file (Fig. 7B).

## Operating instructions

Before switching on the system, make sure that:

- the formatted SD card is inserted correctly (Fig. 5B),
- the program is uploaded (Fig. 6),
- the RTC time is set (Fig. 7).

Switching on the system:

- Connect the joined batteries and the solar panel to the LiPo Rider power supply regulator (“SOLAR” and “BAT” connector of the LiPo Rider).
- Test the batteries using the switch located on the solar panel regulator: if the four LEDs are lit, the battery is full.
- Switch on the e-RFIDuino station: on/off switch on the LiPo Rider solar panel controller.
- Check that the red LED on the Arduino board is lit; if so, it means that the board is now powered.

**Fig. 8:**
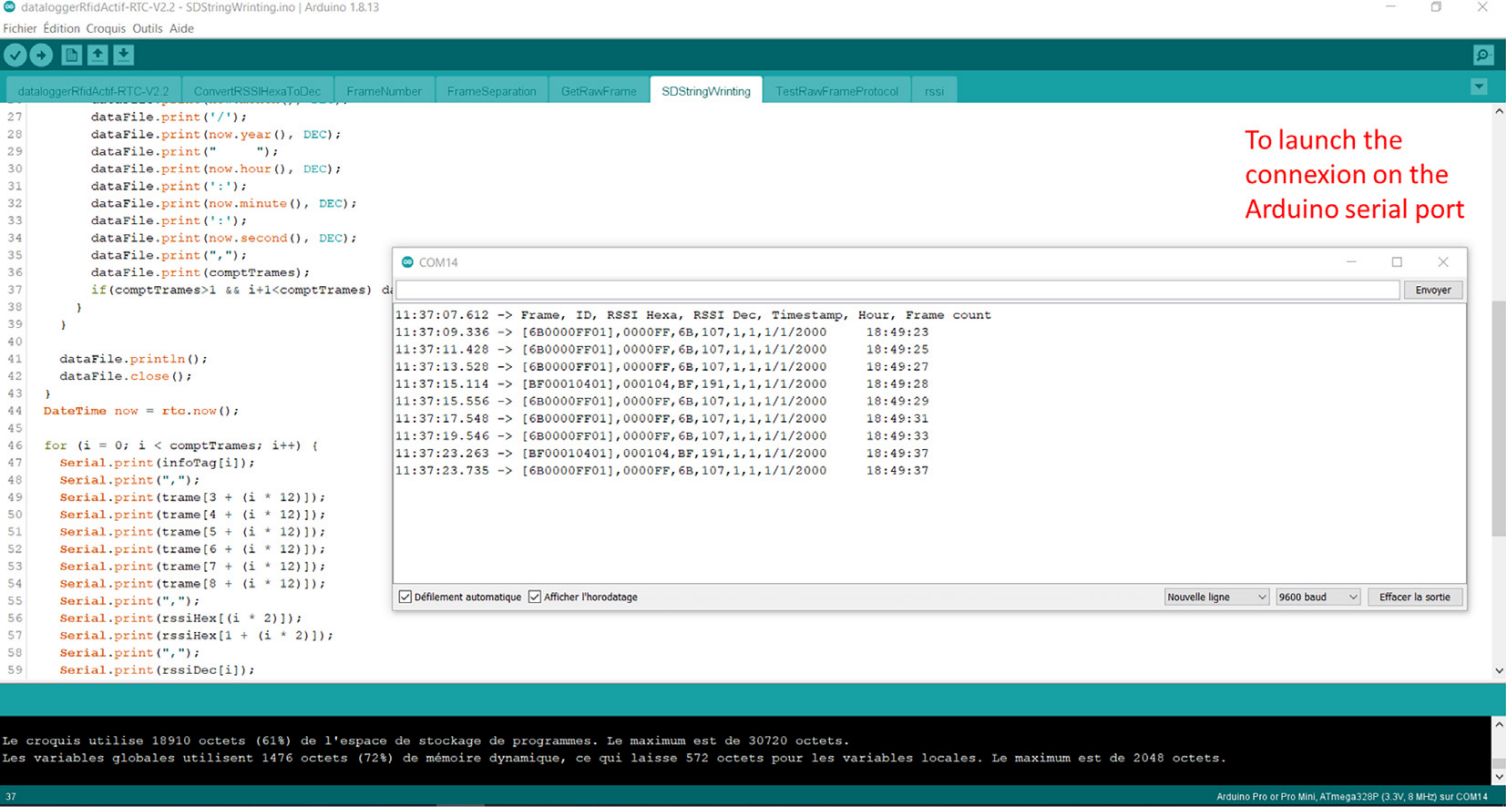
Checking that the system is working properly.

Checking that the system is working properly:

- Test the system by placing an RFID tag close to the antenna; check that the green light on the Arduino module flashes and/or the SD reader light flashes: this means that the reader has detected the tag and that the microcontroller has recorded the tag identifier and the time on the microSD card.
- Check that the recording is correct, that the ID number corresponds, and that the time stamp is correct, by 1) first switching off the power supply (switch on/off the solar panel control board), and 2) removing the SD card.
- The system can also be checked directly through the serial port by connecting the USB-Series FTDI GT1125 converter to the Arduino (Fig. 8). At this stage, it is important that the switching voltage of the FTDI GT1125 converter is set to 5 V (Fig. 6); otherwise, there is a risk of damaging this component.

Data recovery and station maintenance:

- Switch off the e-RFIDuino station: on/off switch on the LiPo Rider solar panel controller;
- Remove the SD card and retrieve the data from the computer;
- Check the data and store it;
- Format the SD card and insert it correctly;
- Check the RTC drift and set the RTC time if necessary (Fig. 7, Fig. 8);
- Switch on the system (see “Switching on the system” procedure).

## Validation and characterization

**Fig. 9:**
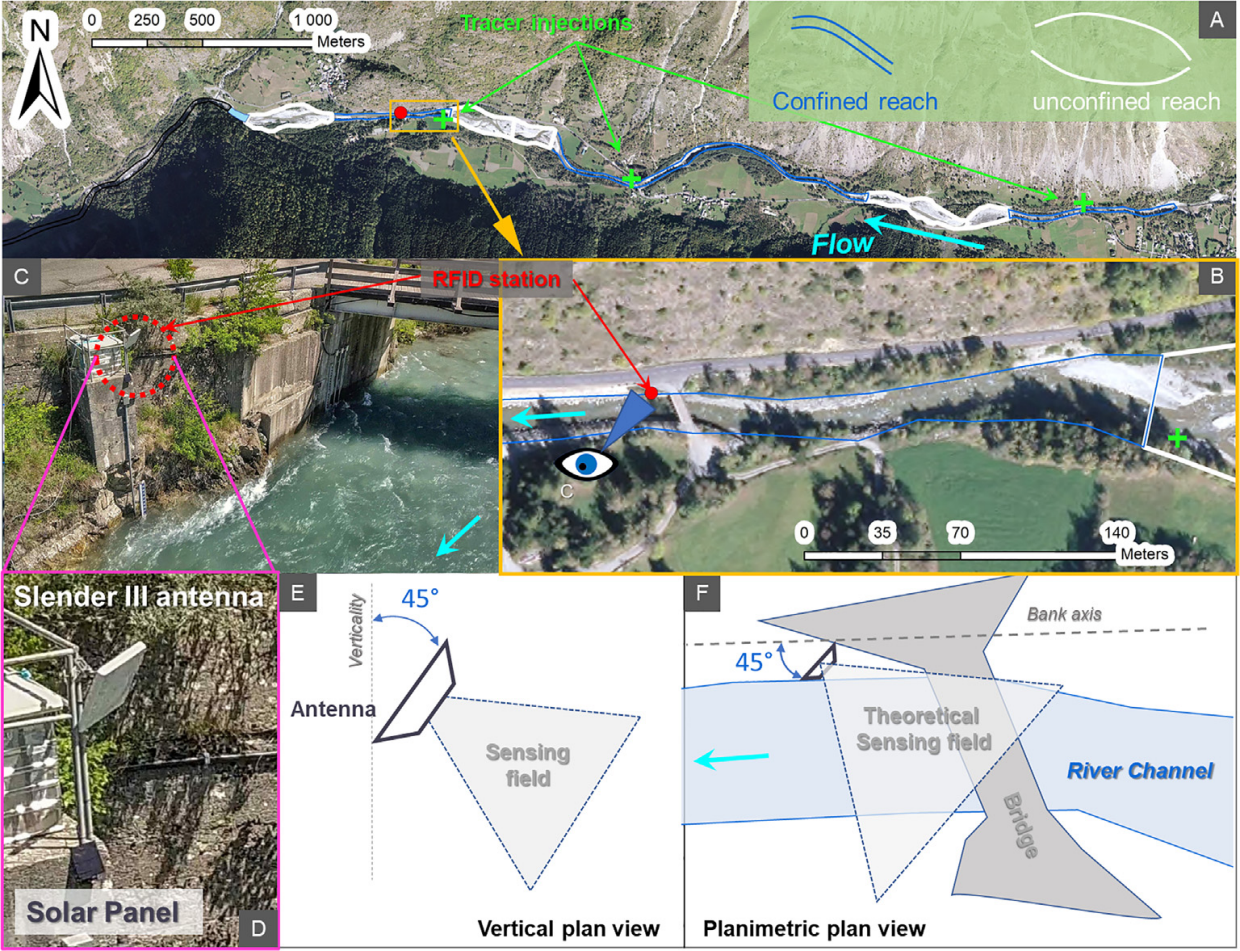
The e-RFIDuino Station installed on the Séveraisse River. (A) the river channel with (B) a straight, narrow, and confined reach was selected. (C) The system was located on the right bank with (D) the Slender III antenna and the solar panel facing south. (E) and (F) show the antenna’s sensing field.

The e-RFIDuino station was deployed on 28 May 2020 along a reach of the Séveraisse river [15] downstream from La Chapelle-en-Valgaudémar in the Ecrins Massif (SE French Alps), and was removed on 26 October 2020 (Fig. 9). To ensure the good detection of tracked objects injected upstream, the e-RFIDuino station was installed along a straight, narrow, and confined channel of 12 m in width. The Slender III antenna, which has a semi-directional sensing field, was fixed on the right bank with an angle of approximatively 45° between the antenna nadir and the vertical axis, and with an angle of 45° between the antenna plan and the river channel axis. In that location, about 3 m above the channel bottom, the e-RFIDuino station cannot be damaged by river flow like it has been previously experimented [13], [14].

**Fig. 10:**
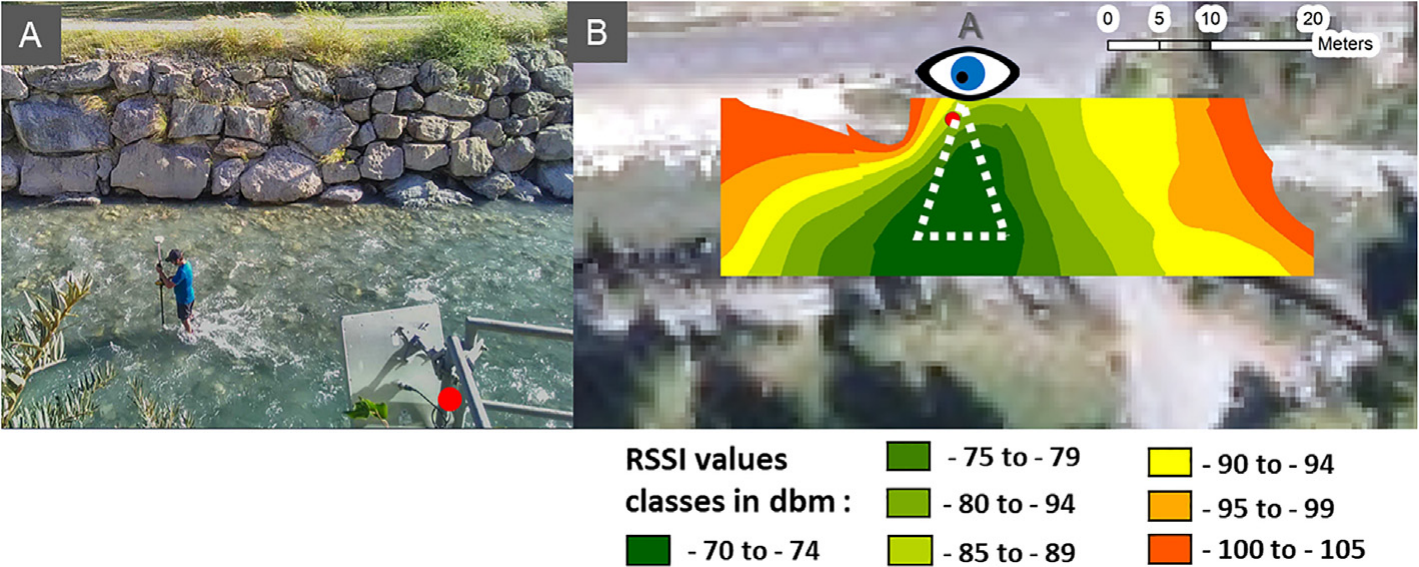
Operator during the on-site determination of the antenna sensing field (A) and the received signal strength indication spatially determined from 30 sampling points (B).

To determine the on-site delimitation of the antenna sensing field, an operator walked backward, forward, and across the river channel with a verification COIN ID tag attached to a GPS pole that was also equipped with a Trimble GeoHX GPS (Fig. 10A). For about 30 points, the operator maintained the COIN ID at the bottom of the channel between the largest stones and geolocated the point while a second operator recorded the COIN ID signal at the e-RFIDuino station. Each placement was maintained for several minutes to ensure that the transponder signal was recorded over at least one full minute. The 30 sampling points were then used to spatially determine the receiver signal strength across the antenna sensing field (Fig. 10B).

This field survey showed that the COIN ID tag signal could be sensed over a linear distance of 60 m. It is important to note that using transponder models with higher power emission, the sensing field and the linear distance of detection would be longer.

**Fig. 11:**
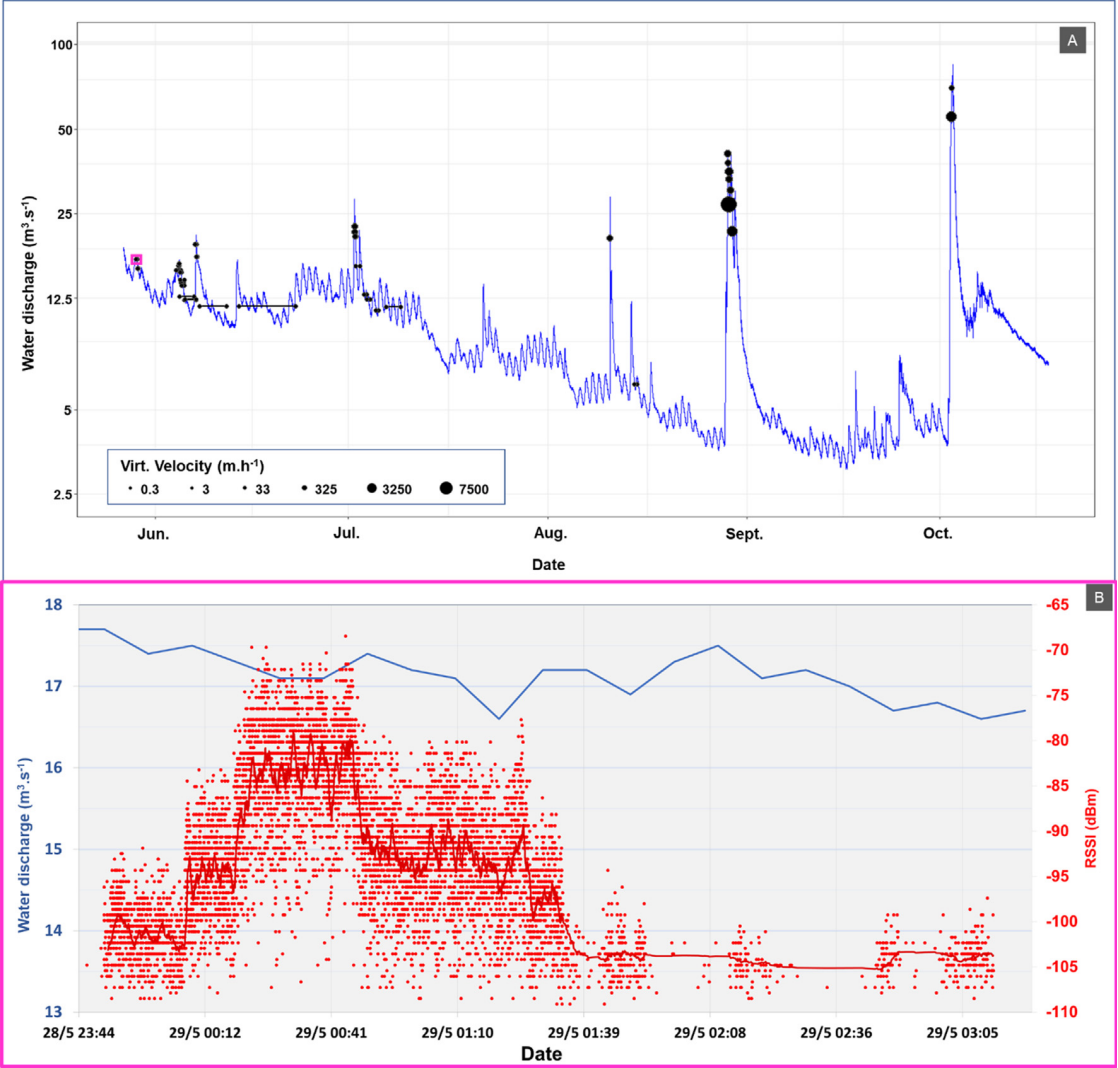
Example of the monitoring of tracked particle passages during the 2020 field campaign. (A) Date and mean velocity of tracer’s passages with regard to water discharge chronic. (B) Focus on a single tracer detailed signal at its passage. Notes: in (A) Black dots indicate timestamp of entry and exit within the antenna sensing field and have a size function of the trace virtual velocity and are scattered at the mean water discharge observed during their passage. Black horizontal lines indicate a long stay within antenna sensing field. Purple scare highlight in hydrological chronic the passage of the tracer detailed in (B). In (A) and (B) blue lines indicates water discharge values reported on left-side ordinate axis. In (B) red dots indicate the Received signal strength indication (RSSI) in dBm reported on the right-side ordinate axis of a single beacon signal detection. The red line represents the rolling average in dBm reported on the right-side ordinate axis and based on 100 observations. (For interpretation of the references to colour in this figure legend, the reader is referred to the web version of this article.)

During the monitoring period, the e-RFIDuino station was powered by a solar panel and two batteries. Although the solar panel had been turned upside down when we arrived on site in August, the e-RFIDuino station was still operating, and it detected a control transponder as soon as it entered the antenna sensing field. Between the 28 May 2020 and the 26 October 2020, the e-RFIDuino station recorded the passage of 31 tracers equipped with COIN ID tags during contrasting flood events (Fig. 11A). Each of the individual tracer sensing durations was divided by this longitudinal distance to measure virtual velocity of individual particles [16].

For our currently performed trials, we report a 100% detection rate of tracked objects, even though they were transported in the bottom of the river flow channel and immersed at depths estimated to be up to 1 m during some of the monitored floods. This detection rate is higher than the detection rate obtained so far with RFID station using PIT tags, ranging from 16% to 39% [12], [14].

This detection rate is defined as the number of tracked objects that were located upstream of the e-RFIDuino station once it was installed and that were detected before e-RFIDuino station removal divided by the number of tracked objects that were located upstream of the e-RFIDuino station once it was installed and that were recovered downstream of the e-RFIDuino station by pedestrian operators or UAV survey. Tracked objects located downstream were recovered during three field campaigns lasting a week, which took place in July, August, and October, and exhibited tracer recovery rates between 60% and 70%, which demonstrate the very good detection rate of the stationary antenna.

The flood events observed on the flow series cover a large span of instantaneous discharges ranging from 12.5 m^3^.s^−1^ up to 77 m^3^.s^−1^, and corresponding with a maximum water depth of 1.15 m at 77 m^3^.s^−1^. This record demonstrates the e-RFIDuino station’s capacity to sense tracked particles transported under most of the hydraulic conditions observed within this reach.

In addition, the records of the detailed RSSI variations of individual tracers provide information on their movement and rest phases (Fig. 11B). This information is relevant for the analysis of the particle virtual velocity values, which ranged from 0.3 m.h^−1^ to 7548 m.h^−1^, has it allows to count and time the movement and rest phases.

These encouraging results offer new perspectives for the more-detailed exploration of particle mobility and bedload processes, and how they vary according to flood events and the weather conditions generating them (snowmelt periods, short and very intense summer storm events, intense prolonged rainfall events), with the aim of better understanding the relationships between water and sediment fluxes.

Furthermore, they demonstrate the efficiency and potential of the e-RFIDuino station for monitoring tagged objects in harsh off-grid environments.

## Future developments

While the e-RFIDuino station was developed to function in an off-grid manner, a potential improvement would involve data transmission over the mobile telephone network, thus reducing the field effort and time required to retrieve data, as well as providing real-time monitoring that could be used to trigger field acquisitions at the most important times. With a supplementary module to transmit data to the mobile telephone network, further optimization of the power supply and consumption may be needed, but this is likely to be feasible.

Although the RFID system used in this study functioned with a proprietary communication protocol, the e-RFIDuino hardware could be customized to operate with other RFID systems, using nonproprietary communication protocol for instance. A small modification of software libraries would probably be needed to adapt the new RFID system frame format.

## Declaration of Competing Interest

The authors declare that they have no known competing financial interests or personal relationships that could have appeared to influence the work reported in this paper.
