# Supplementary material for: The e-RFIDuino: An Arduino-based RFID environmental station to monitor mobile tags
Source: HardwareX. 2021 Jun 12;10:e00210. doi: 10.1016/j.ohx.2021.e00210 (PMC9123422; doi:10.1016/j.ohx.2021.e00210)
Supplement: Supplementary data 1 [file mmc1.docx]

**Supplementary material: Table of acronyms**

| **Acronym** | **Signification** |
| --- | --- |
| *RFID* | Radio Frequency Identification |
| *PIT* | Passive Integrated transponders |
| *LF* | Low Frequency |
| *Tag* | Transponder |
| *a-UHF tag* | Active ultra-high frequency transponders |
| *COIN ID* | Name of the a-UHF tag model used |
| *RSSI* | Received Signal Strength Indication |
| *UAV* | Unmanned Aircraft Vehicle |
| *RTC system* | Real Time Clock system |
| *W* | Watt |
| *dB* | Decibel |
| *dBm* | Decibel per MilliWatt |
| *Q* | Water discharge in cubic meter per second |
